# Complete mitochondrial genome of the sharp-snouted pitviper *Deinagkistrodon acutus* (Reptilia, Viperidae)

**DOI:** 10.1080/23802359.2019.1660593

**Published:** 2019-09-05

**Authors:** De-Qing Wang, Li-Li Pan, Dian-Cheng Yang, Liang-Liang Dai

**Affiliations:** College of Life and Environment Sciences, Huangshan University, Huangshan, P. R. China

**Keywords:** *Deinagkistrodon acutus*, wild-type, mitochondrial genome, phylogeny

## Abstract

The sharp-snouted pitviper, *Deinagkistrodon acutus*, belongs to the monotypic genus *Deinagkistrodon* of the family Viperidae. It is one of the important species of snakes for its commercial and medicinal value. Complete mitochondrial genome sequence of the *D*. *acutus* was sequenced by next-generation sequencing technology. The total mitochondrial genome is 17,538 bp in length, containing 13 protein-coding genes, 22 tRNA genes, 2 ribosome RNA genes, and 2 control regions. Most of the genes of *D*. *acutus* are encoded on the H-strand, except for the ND6 subunit gene and eight tRNA genes which distribute on the L-strand. Phylogenetic reconstruction result indicated that our newly determined mitochondrial genome sequence could meet the demands. The complete mitochondrial genome sequence presented here will be useful to study the evolutionary relationships and genetic diversity of *D*. *acutus*.

The sharp-snouted pitviper, *Deinagkistrodon acutus*, belongs to the monotypic genus *Deinagkistrodon* of the family Viperidae. It is one of the important commercial and medicinal species of snakes due to its large body and venom. It is distributed in southern China, from the west of the Taiwan, Zhejiang, and Fujian Province to the east of the Chongqing Municipality and Guizhou province, northern Vietnam and southwest Laos (Zhao 2006; Huang et al. 2007). *Deinagkistrodon acutus* is a young population with low-genetic diversity, geographically, the Poyang Lake, Gan River, and the Wuyi Mountains divided the whole population into two main lineages (western and eastern). The Huangshan population is derived from both main lineages and is the highest nucleotide diversity units (Huang et al. 2007). Three mitochondrial genomes of this species had determined (Yan et al. 2008), only one described the sample location, the article is unpublished. Here, we determined and reported the mitochondrial genome of wild-type *D. acutus* from Huangshan.

The specimen of *D*. *acutus* was collected from Qimen County, Huangshan, Anhui, China, and was preserved and deposited in the Museum of Huangshan University (Voucher number: HUM2018032). Complete mitochondrial genome sequence of this specimen was sequenced by next-generation sequencing technology (Illumina Hiseq 2500) at Shanghai Majorbio Bio-pharm Technology Co., Ltd (Shanghai, China). Genome sequences were picked out and assembled by the software SOAPdenovo v2.04 (Luo et al. 2012) and MITObim v1.6. The positions of tRNA and rRNA genes were predicted by the MITOS (Bernt et al. 2013) and the locations of protein-coding genes were identified by comparing with the homologous genes of conspecific and other closely related species.

The complete mitochondrial genome of *D*. *acutus* (Genbank accession number MK450437) was sequenced to be 17,538 bp in length with overall base composition as follows: A (32.5%), T (25.3%), C (29.4%), and G (12.8%), of which the percentage of G + C is 42.2%. The genome consists of 13 protein-coding genes, 22 transfer RNA (tRNA) genes, 2 ribosomal RNA (rRNA) genes, and 2 control regions (D-loop). Most of the *D*. *acutus* mitochondrial genes are encoded on the H-strand except for the ND6 gene and eight tRNA genes, which are encoded on the L-strand. Eight of the 13 protein-coding genes (COII, ATPase 8, ATPase 6, COIII, ND4, ND5, ND6, and CYT b) initiate with ATG as start codon, while COI and ND4L genes are initiated by GTG, ND2, and ND3 genes begin with ATT and ND1 starts with ATA. Seven protein-coding genes (COI, ATPase 8, ATPase 6, ND4L, ND4, ND5, and ND6) end with complete stop codons (TAA, AGA, and AGG) and the others end with T as the incomplete stop codons. The 12S rRNA (903 bp) and 16S rRNA (1483 bp), are located between the tRNA-Phe and tRNA-Lue genes and separated by the tRNA-Val gene.

The whole mitochondrial genome sequence of *D*. *acutus* determined in this study and together with other 17 closely related species from GenBank to perform phylogenetic analysis. A maximum likelihood (ML) tree was reconstructed based on the dataset by online tool RAxML (Kozlov et al. 2018). Phylogenetic analysis result indicated that our newly determined mitochondrial genome sequence could meet the demands and explain some evolution issues (Figure 1).

**Figure 1.**
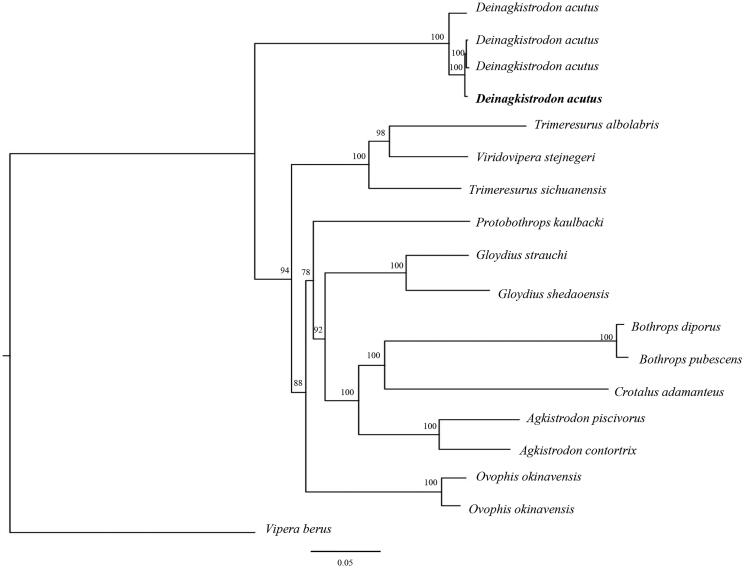
A maximum likelihood (ML) tree of the *Deinagkistrodon acutus* in this study and other 17 closely related species was reconstructed based on the dataset of the whole mitochondrial genome by online tool RAxML. The numbers above the branch meant bootstrap value. Bold black branches highlighted the study species and corresponding phylogenetic classification. The analyzed species and corresponding NCBI accession number is as follows: *Deinagkistrodon acutus* (KT225463), *D. acutus* (DQ343647), *D. acutus* (EU913476), *D. acutus* (MK450437), *Trimeresurus albolabris* (NC_022820), *Viridovipera stejnegeri* (FJ752492), *Trimeresurus sichuanensis* (KT266810), *Protobothrops kaulbacki* (KY695463), *Gloydius strauchi* (MF523224), *G. shedaoensis* (KT726957), *Bothrops diporus* (MG182599), *B. pubescens* (MG182598), *Crotalus adamanteus* (MH626511), *Agkistrodon piscivorus* (DQ523161), *A. contortrix* (KY747498), *Ovophis okinavensis* (AB175670), *O. okinavensis* (LC073749), *Vipera berus* (MF945570).
